# Exercise Intensity Modulation of Hepatic Lipid Metabolism

**DOI:** 10.1155/2012/809576

**Published:** 2012-04-02

**Authors:** Fábio S. Lira, Luiz C. Carnevali, Nelo E. Zanchi, Ronaldo VT. Santos, Jean Marc Lavoie, Marília Seelaender

**Affiliations:** ^1^Laboratory of Exercise Biochemistry and Physiology, Health Sciences Unit, University of Southern Santa Catarina, 88806-000 Criciúma, SC, Brazil; ^2^Cancer Metabolism Research Group, Institute of Biomedical Sciences, University of São Paulo, 05508-900 São Paulo, SP, Brazil; ^3^Institute of Biomedical Science, University of São Paulo, 05508-900 São Paulo, SP, Brazil; ^4^Departamento de Biociências, Universidade Federal de São Paulo, Campus Baixada Santista, Brazil; ^5^Department of Kinesiology, University of Montreal, Montreal, P.O. Box 6128, ON, Canada H3C 3J7

## Abstract

Lipid metabolism in the liver is complex and involves the synthesis and secretion of very low density lipoproteins (VLDL), ketone bodies, and high rates of fatty acid oxidation, synthesis, and esterification. Exercise training induces several changes in lipid metabolism in the liver and affects VLDL secretion and fatty acid oxidation. These alterations are even more conspicuous in disease, as in obesity, and cancer cachexia. Our understanding of the mechanisms leading to metabolic adaptations in the liver as induced by exercise training has advanced considerably in the recent years, but much remains to be addressed. More recently, the adoption of high intensity exercise training has been put forward as a means of modulating hepatic metabolism. The purpose of the present paper is to summarise and discuss the merit of such new knowledge.

## 1. Introduction

Lipid metabolism involves numerous pathways that are, at least partly, interdependent. The lipid available for liver uptake may derive from the diet [1] or from mobilisation of fatty acids (FAs) from the adipose tissue, followed by the transport in the circulation, [2], which requires specific transporters such as albumin, while diet lipids in the form of triacylglycerol (TG) are transported by chylomicra and very low density lipoprotein (VLDL). In the liver, specific transporters (FAT and L-FABP) are involved in the uptake and intracellular traffic of these molecules [3, 4]. The hepatocyte then carries out TG hydrolysis to diacylglycerol (performed by microsomal lipase) and then to fatty acids, which are then activated and combined with coenzyme A, allowing their transport into the reticulum luminal space by intraluminal carnitine acyltransferase, where they are again esterified by TG diacylglycerol acyltransferase 2 [5], and become a part of nascent hepatic VLDL, or are stored within lipid droplets. Long-chain fatty acids (LCFA) deriving from exogenous sources or from intracellular pools may conversely be totally or partially oxidised by the hepatocyte mitochondria, a process that requires the action of an enzyme system, the carnitine palmitoyltransferases, and the channelling of the fatty acyl to either ketone body production or to B-oxidation. Finally, other possible fates of LCFA include the modification of the molecule, yielding, for instance, cholesterol and the incorporation into components of the cells, as into the membrane phospholipids [6]. The final fate of LCFA in the liver depends on a plethora of factors, including the quantity and type of fatty acid, hormonal regulation, contribution of innervation, and cell communication within the organ, to name a few.

Indeed, lipid metabolism in the liver is very complex, an aspect which is illustrated by the synthesis of VLDL. Most of the triacylglycerol (TG) recruited for the assembly of VLDL in the secretory apparatus of the hepatocyte is mobilised by lipolysis of preexistent cytosolic TG pools, followed by reesterification [7]. The assembly of VLDL in hepatocytes requires the microsomal triacylglycerol transfer protein (MTP), which transfers lipids, particularly TG, between membranes, and it is known to transfer TG to nascent apolipoprotein B (apoB) *in vivo* [3]. Some of the TG, however, is returned to the cytosolic pool in a process that is stimulated by insulin and inhibited by MTP [6]. As mentioned, every step of the VLDL synthesis is modulated by the physiological status of the organ.

It is well established that physical inactivity is related with excess plasma triglyceride (TG) concentration, which contributes, at least partially, to increased disease risk, as appearing in association with atherosclerosis, fatty liver, diabetes, and obesity [8, 9]. On the other hand, chronic exercise training has been shown to present favorable effects on plasma lipid profile [10–12]. In this context, chronic exercise, especially moderate aerobic exercise (50%–75% VO_2peak_) has been considered one of the best nonpharmacological strategies for preventing and treating cardiovascular diseases, Position Stand American College of Sports Medicine 2011 [13].

Recently, high intensity exercise (>80% VO_2peak_) has been shown to markedly affect plasma lipid metabolism [14, 15]. In addition, the higher energy expenditure achieved by associating volume and intensity seems to promote more prominent changes in liver lipoprotein and oxidative metabolism [14, 16]. Considering the relevance of the liver for lipid metabolism regulation, the purpose of this paper is to summarise the specific effects of moderate and high intensity exercise on hepatic lipid metabolism.

## 2. Exercise Intensity and Plasma Lipid Profile

Several studies concerning the effects of exercise on plasma lipid profile have suggested that there is an intensity threshold for eliciting changes in these parameters (see Table 1). However, is there in fact a direct relationship between exercise intensity and lipid profile?

Significant improvements in HDL-cholesterol levels were reported in volunteers who exercised at 75% of maximal heart rate for 12 weeks, but no changes were observed in those who exercised at 65% of maximal heart rate [17]. In addition, Ferguson et al. [18] reported that after an aerobic exercise session, only the subjects with higher energy expenditure showed a reduction in TG and an increase in HDL-c concentrations, leading forth to the hypothesis of the existence of a threshold of energy expenditure associated with changes in lipoprotein profile. These data indicate that moderate intensity acute exercise that induces energy expenditure over 1,100–1,500 kcal, performed at approximately 70% of maximal oxygen consumption, has a greater effect on HDL cholesterol when compared with acute exercise with low energy expenditure. Therefore, intensity seems to be an important modulator of lipid profile [18].

Aellen et al. [2] reported that after 9 weeks of aerobic or anaerobic training, only aerobic exercise performed below lactate threshold was capable of inducing beneficial effects upon lipoprotein profile, while the anaerobic protocol (isocaloric expenditure energy) failed, especially in regard to the antiatherogenic lipoproteins. However, in a recent study, Tsekouras et al. [19] examined the effect of high intensity intervals of aerobic training (2 months of supervised high-intensity interval training, 3 sessions/wk; running at 60 and 90% of peak oxygen consumption in 4 min intervals for a total of 32 min) on VLDL-TG secretion in men. They reported that subjects who had ran on the treadmill for 8 weeks at 90% VO_2peak_ presented a reduced rate of VLDL-TG secretion, suggesting that even high intensity exercise may induce changes in lipid profile. Thus, not only exercise intensity, but the effect of exercise intensity plus energy expenditure appear to modulate the rate of VLDL-TG secretion, which should be taken into consideration when designing or studying the exercise training effects focusing on promoting benefits upon lipoprotein profile.

## 3. Effect of Diet Manipulation and Exercise on Lipid Profile

It has been long recognised that diet manipulation in combination with exercise training is capable of modulating lipid profile. However, an important question to be considered is what is the effect of diet manipulation during different exercise intensities? In order to answer the first question, Maraki et al. [20] showed that even a low intensity aerobic exercise session (30% VO_2peak_), that theoretically does not induce a hypotriacylglycerolaemic effect (<2 MJ), when combined with caloric restriction is capable of promoting one such response. On the other hand, we recently tested the effects of ingesting different CHO content diets and high intensity exercise on lipid profile [14]. Our aim was to compare the effects of high intensity exercise (~90% VO_2max_) and a dietary intervention (control, low, and high carbohydrate content) on blood lipid profile (VLDL, HDL cholesterol, LDL cholesterol, and total cholesterol). We hypothesised that high intensity exercise combined with carbohydrate supplementation could exert beneficial effects upon lipoprotein profile in healthy men. Although several studies showed that the reduction in TG and the increase in HDL-c concentrations promoted by exercise are dependent on a high energy expenditure, our results demonstrated that acute high intensity exercise, with low energy expenditure (in the absence of dietary interventions e.g., control group) was able to reduce LDL-c and total cholesterol levels. Having found out that acute high intensity exercise (~90% VO_2max_) reduces lipoprotein levels, even when associated with low energy expenditure, our group sought to investigate the effects of acute supramaximal intensity exercise (approximately 115% VO_2max_) on lipoprotein metabolism [21]. In this study, the acute exercise session generated 50% less energy expenditure than the previous study [14]. The data showed that a supramaximal exercise session has no significant effects on lipid metabolism [21]. Therefore, we suggest the existence of an “energy expenditure threshold” to induce changes in blood lipid levels.

Collectively, these data reinforce the hypothesis that there is a balance between intensity and the level of energy expenditure (that can be improved, especially during low intensity exercise, when reinforced by diet manipulation) that is able to induce changes in the lipoprotein profile, with special regard to VLDL secretion.

## 4. Resistance Exercise and Lipid Metabolism

Resistance exercise (mainly acute) has also been reported to affect lipid metabolism. Wallace et al. [23] showed that moderate acute resistance exercise (73% of 1 RM) induced favorable modifications of lipid profile, increasing HDL-c and its subfractions (HDL2 and HDL3), when compared with high intensity resistance exercise (92% 1 RM). This result can be a consequence to the differences between volume loading (high intensity exercise with low volume loading −7.04 = sets × repetitions × weight, and moderate intensity high volume loading −31.13 = sets × repetitions × weight). Thus, total energy expenditure may, at least partly, determine lipid metabolism modification following physical activity. As a potential alternative explanation, Tsekouras et al. [24] demonstrated that acute resistance exercise does not affect the rate of hepatic secretion of VLDL-TG, but increases VLDL-TG plasma clearance rate by 26% as compared with the rest. Yet, the mean residence time of VLDL-TG in the circulation was significantly shorter after exercise than during rest (113 min after exercise than 144 min in rest). In fact, earlier, Magkos et al. [26] observed that acute resistance exercise was more efficient than aerobic exercise in increasing the clearance of VLDL and TG, and in decreasing the mean residence time of these lipoproteins in the circulation, suggesting a particular regulatory mechanism elicited by acute resistance exercise. Nonetheless, it remains unknown if such modification constitutes a fingerprint modification elicited in an intensity specific manner on lipid profile.

Magkos et al. [15] recently described possible routes of VLDL and TG removal from plasma, including hydrolysis by lipoprotein lipase (LPL) and possibly also by hepatic lipase, transfer of TG to other lipoproteins (e.g., HDL) via neutral lipid exchange, conversion of VLDL to lipoproteins of higher density, for example, intermediate-and low-density lipoproteins (IDL and LDL, resp.), as well as removal of the whole VLDL particle from plasma via interaction with hepatic and/or peripheral receptors [19, 26, 27]. On the other hand, it is well documented that regular physical exercise is able to induce an augmentation of LPL gene expression and activity in the skeletal muscle [27, 28], resulting in decreased plasma TG content, which is also linked with decreased liver VLDL output [11, 12, 22]. These observations suggest a relationship between the increased catabolic rate of VLDL and TG during the early phase of recovery and repletion of the intramuscular TG *pool*. This is further supported by the transient increase in the transcription rate of muscle LPL described by Pilegaard et al. [29] 1 h after a 60–90 min of exhaustive knee-extensor exercise. Thus, whereas VLDL and TG turnover rate returned to its basal value 2-3 h after exercise, the peak in muscle LPL mass is reached 8 h after exercise [30]. This may constitute a critical regulatory mechanism elicited by exercise to reduce VLDL and TG levels.

In a recent study [25], we reported evidence that corroborates the hypothesis that acute resistance exercise in a moderate/high intensity (as well as aerobic exercise) may have antiatherogenics effects, particularly throughout lipid profile modulation. The experimental design consisted of five groups that performed acute exercise at different percentages of the one repetition maximum (1 RM); 50%-1 RM, 75%-1 RM, 90%-1 RM, and 110%-1 RM group. The total volume (sets × reps × load) of the exercise was equalised. We demonstrated that acute resistance exercise may induce changes in lipid profile in a intensity-specific manner, taking into consideration that in such study although the intensity seems to be an important factor, the “threshold” must to be respected to induce benefits on lipid profile (low to moderate at 50% and 75% 1 RM intensities seems to be more proper to induce benefits on lipid profile than high-intensity at 90% and 110% 1 RM exercise). Thus, the fitness professional should consider intensities ≤ or = 75% of 1 RM when prescribing resistance training programs aimed at improving lipid profile. Chronically applied, such response might possibly yield greater benefits in increasing HDL-c and diminishing VLDL and TG lipoproteins, when compared with other strength training intensities. However, future studies are necessary to unveil potential mechanisms.

## 5. Exercise Intensity and Lipid Oxidation in the Liver

It is well documented that there is an important lipid profile adaptation, especially in regard to VLDL secretion, promoted by both acute and chronic exercise [15, 19, 26]. Such adaptation is usually related to an increased fatty acid delivery and oxidation in skeletal muscle promoted by LPL and the carnitine palmitoyltransferase (CPT) system adaptation even under intermittent high-intensity training [31]. However few studies have been performed to analyse the adaptations imposed by such stimuli upon hepatic lipid oxidation.

Observational studies in human suggest that increased habitual activity is inversely associated with intrahepatic TG content [32] and endurance training in animals reduces liver fat accumulation [33, 34]. However it is not clear whether physical exercise has a direct impact on the enzymatic and molecular processes regulating lipogenesis and/or lipid oxidation in liver. Yasari et al. [35], showed that 8 weeks of treadmill exercise training (~60–70% VO_2max_) were able to downregulate the gene expression of SCD-1 (stearoyl-Coa desaturase-1), a rate-limiting enzyme in the biosynthesis of saturated-derived monounsaturated fat that are the major constituents of VLDL-TG in 2-week high fed rats. It is well documented that SCD-1 inhibition reduces lipogenesis and enhances hepatic fatty acid oxidation [36].

In a recent study performed by our group [37], we observed that 8 weeks of treadmill moderate training (~60% VO_2max_) increased CPT (carnitine palmitoyltransferase) complex maximal activity in the liver and prevented hepatic steatosis in trained tumour-bearing rats, reinforcing the hypothesis that an environmental factor such as exercise is able to optimise hepatic lipid oxidation. In agreement with such finding, Rector et al. [34, 38] reported that attenuation of hepatic steatosis in response to voluntary exercise training is associated with both increased hepatic fatty acid oxidation and likely reduced fatty acid synthesis, as indicated by reductions in key protein intermediates.

Malonyl-CoA is the first committed intermediate in the lipogenic pathway and is also an inhibitor of carnitine palmitoyltransferase-1 (CPT-1) the enzyme that controls the transfer of cytosolic long-chain fatty acyl CoA (LCFA CoA) into mitochondria. Therefore, CPT-1 activity can be limiting for fatty acid oxidation and ketogenesis in liver [39]. The decline in liver malonyl-CoA has been postulated to be responsible for the increase in blood ketone production during and after exercise [5]. Acetyl-CoA carboxylase (ACC) is the enzyme responsible for malonyl-CoA synthesis and its activation is allosterically regulated by citrate and inhibited by palmitoyl-CoA [40] and by AMPK (5′-AMP-activated protein kinase) phosphorylation [41].

 Due to an increase in plasma TG and VLDL-TG concentration, it is likely that palmitoyl-CoA would have been elevated in the hepatocytes, bearing in mind that it represents an allosteric inhibitor of ACC and hence of malonyl-CoA synthesis [40, 42].

## 6. Exercise Intensity and Hormone Profile

Several studies show a clear effectiveness imposed by training intervention upon hepatic lipid content and a significant adaptation of hepatic metabolism to regular physical activity [43]. This adaptation is subject to a refined control by endocrine parameters, specially the insulin/glucagon ratio, and the stress promoted by exercise is known to modulate plasma concentrations of these hormones, which could be involved in the modulation of pathways controlling in the transcriptional regulation of hepatic genes after acute exercise [44]. Glucagon and insulin balance is an important contributor to the increase of hepatic fat oxidation during exercise. In response to moderate-intensity exercise, the secretion of glucagon and insulin from the pancreas generally increases and decreases, respectively [45].

During acute physical activity, an important interaction between the liver, muscle, and adipose tissue occurs, as to provide and maintain adequate blood levels of glucose, free fatty acids (FFAs), and consequently, ATP levels for the contracting muscle [46]. In addition, it is well known that during exercise, insulin levels are decreased allowing the mobilisation of the supracited fuel substrates supporting skeletal muscle contraction [47]. However, more recently Rector et al. [38], studying the Otsuka Long-Evans Tokushima Fatty rat, a common model of obesity, hepatic steatosis, and type 2 diabetes, examined the transition from free wheel access to voluntary running for 16 weeks (in order to prevent the development of hepatic steatosis) to a sedentary condition. After the cessation of daily exercise (5–173 h), no changes occurred in body weight, fat pad mass, food intake, serum insulin, hepatic triglycerides, or in the exercise-suppressed hepatic stearoyl-CoA desaturase-1, but complete hepatic fatty acid oxidation and mitochondrial enzyme activities were reduced in the rats that remain sedentary by 173 hours. A new question that arises from this topic is how slowly would be the transition of an active state (hyperkinesia) to a sedentary state in humans? Is it possible to avoid these deleterious effects upon the liver after the cessation of physical activity?

In another study, a recent question put foward by Noland et al. [48] was “does having an elevated initial oxidative capacity, provided by a genetic model with inherent initial differences in aerobic capacity allow protection against the development of obesity and diabetes?” To investigate this, the authors studied rats with low (LCR) and high (HCR) capacity endurance running fed with a pattern chow diet or a high fat diet (HFD). The authors demonstrated that elevated basal skeletal muscle oxidative capacity and the ability to preserve liver oxidative capacity may protect HCR rats from HFD-induced obesity and insulin resistance. Thus, renewed questions regarding insulin and the liver are of interest.

On the other hand, the increase in glucagon enhances the oxidation of NEFAs by stimulating pathways for fat oxidation within the liver [49], and this increased hepatic fat oxidation promoted by exercise produces energy that fuels gluconeogenesis [50]. The liver may itself modulate glucagon action in relation to exercise, considering that, under glucagon infusion, liver glucose production at rest is higher in trained individuals than in sedentary subjects [51], and also that exercise is able to promote a higher liver sensitivity to glucagon that could be due, at least in part, to an increased glucagon receptor density in liver [52]. The magnitude of these parameters generally increases with greater exercise duration and intensity [49]; however the question resides over the influence of acute and chronic exercise of different intensities upon glucagon secretion and liver metabolism modulation.

Most investigations concerning hepatic regulation during exercise have been performed at moderate intensities, although at higher intensities such regulation may be quite different and interesting. Firstly, the main identifiable difference is that during high intensity exercise (approximately 100% of VO_2max_) catecholamines may increase by 10- to 15-fold, while arterial glucagon may increase, remain the same, or even decrease [53]. Secondly, circulating glucose levels often increases at high-intensity training and this may prevent the fall or even lead to a increase in insulin levels [54], suppressing glucagon effects, but not affecting catecholamine's function considerably [55, 56]. In fact, earlier studies performed by Galbo [54] and Marker et al. [57] showed that glucagon response to high-intensity exercise was attenuated and led to impairment of liver glycogen breakdown. Furthermore, although catecholamines secretion is higher during high-intensity exercise (74% VO_2max_) in comparison with moderate-intensity exercise (41% VO_2max_), several earlier studies performed in humans showed that the attenuation of sympathetic nerve activity [58], along with experiments with liver transplant patients [59] does not affect the glucose response during a high-intensity exercise. Therefore, exercise performed at high-intensity or for a long duration may be modulating different responses.

 The existence of energetic stress in liver during exercise is evidenced by the modulation of important metabolic pathways in this organ [60]. Exercise under moderate-/high-intensity leads to exhaustion acknowledged to increase glucose production by the liver due to lower circulating glucose levels. Hence, cAMP concentration decreases and AMP-to-ATP ratio increases sufficiently to activate hepatic AMP-activated protein kinase (AMPK) [43, 61]. Berglund et al. [43] recently demonstrated that glucagon exerts a critical regulatory role in the liver, stimulating pathways linked to lipid metabolism *in vivo* and showed that activation of glucagon receptors is implicated in the pronounced transcriptional regulation of hepatic genes after running acute exhaustion exercise at 20 m/min in rats. Glucagon action includes AMPK and p38 mitogen activated protein kinase-dependent activation of peroxisome proliferator-activated receptor-*α* (PPAR*α*) [62]. This finding is important because PPAR*α* is a transcription factor critically required for many aspects of hepatic lipid metabolism [4].

Therefore, studies which showed a favourable lipid profile, including VLDL production by the liver, as induced by exercise, especially those performed until exhaustion or in a higher intensity [19] may be associated with adaptations modulated through glucagon stimulation, although a causal relationship needs still to be proven.

The energetic stress promoted by exercise also elevates plasma catecholamine concentrations, which, via the activation of hepatic adrenergic receptors, may also lead to MAPK activation [63]. However, it is not clear whether catecholamines are involved in the exercise-induced gene expression in the liver [44]. Nevertheless, several studies have been carried out [58, 64–66] to investigate the influence of adrenergic stimulation on glucose and lipid metabolism in the liver. Certainly, we cannot exclude the possibility that hepatic innervation is involved in the activation of hepatic MAPK signalling during exercise and also that the intensity of the stimulus may be an important factor in such modulation.

The information about decreasing liver glycogen content or glucose intermediates during exercise at different intensities is relayed through the afferent activity of hepatic innervation to the central nervous system, contributing to the modulation of the hormonal and consequently metabolic responses to exercise, controlling glucose levels and optimising lipid metabolism in liver.

## 7. Conclusions

In summary, the liver functions as a central manager of lipid metabolism in the organism, by regulating substrate availability to other tissues. The total energy expenditure generated during an exercise session is able to modulate the capacity of the liver to perform this task. However, more studies are necessary to elucidate which are the most important parameters inducing higher hepatic lipid secretion and oxidation during exercise, especially in regard to efforts of high intensity. The answers are very likely to contribute to more precise interventions in the population, contributing in a more consistent manner to health issues.

## Figures and Tables

**Table 1:** Effects of Exercise Intensity on Hepatic Lipid Metabolism in Animal Models and Human.

Reference	Sample	Intensity	Duration	Results
Mondon et al. [22]	Rat males	Moderate intensity 60% VO_2max_	12 weeks	Reduction TG, FFA levels, VLDL-TG secretion was 50% lower in exercise trained rats
Stein et al. [17]	Untrained men	65%, 75% and 85% maximal heart rate	12 weeks	Increases in the HDL cholesterol fractions in the 75% and 85% groups. Significant decreases in LDL fractions in the 75% group
Wallace et al. [23]	Trained men	Moderate (73% of 1 RM) and High intensity (92% 1 RM)	Acute 90 min	Increases HDL-c and its subfractions (HDL2 and HDL3) in moderate when compared to high intensity strength exercise
Aellen et al. [2]	Untrained men	16 trained intensity above and 17 below the anaerobic threshold	9 weeks	Increases in the HDL and HDL2 cholesterol fractions in the below the anaerobic threshold
Lira et al. [11]	Rat males	Moderate intensity 60% VO_2max_	8 weeks	Exercised rats showed reduction of TG, VLDL-TG levels, hepatic tissue TAG content, and lower rate of hepatic VLDL secretion, gene expression of apoB and MTP when compared with control rats
Magkos et al. [15]	Untrained men	80% of peak torque production	Acute 90 min	Resistance exercise lowered fasting plasma VLDL-TG, increased VLDL-TG plasma clearance rate, and shortened the mean residence time of VLDL-TG in the circulation
Tsekouras et al. [19]	Untrained men	60 and 90% of VO_2peak_	8 weeks	High-intensity interval training VLDL-TG concentration was reduced, and this was due to reduced hepatic VLDL-TG secretion rate
Tsekouras et al. [24]	Untrained men	80% of peak torque production	Acute 90 min	Reduced VLDL-TAG concentrations, plasma clearance rate of VLDL-TAG was significantly higher after exercise than rest, and the mean residence time of VLDL-TG in the circulation was significantly shorter. Fasting plasma NEFA and serum beta-hydroxybutyrate concentrations were both significantly higher after exercise than rest
Chapados et al. [12]	Rat males	Moderate intensity 60% VO_2max_	8 weeks	Reduction in liver TG content, reduces VLDL synthesis and/or secretion in fed rats probably via MTP regulation
Lira et al. [14]	Trained men	90% VO_2max_	Acute ~8 min	Total cholesterol and LDL cholesterol were reduced after the exhaustion and 1 h recovery periods when compared with rest periods
Lira et al. [25]	Untrained men	50%, 75%, 90% and 110%-1 RM	Acute ~10 min	The 75%-1 RM group demonstrated TG reduction when compared to other groups. HDL-c concentration was significantly greater after resistance exercise in 50%-1 RM and 75%-1 RM when compared to 110%-1 RM group
Lira et al. [21]	Trained men	115% VO_2max_	Acute ~4 min	There were no significant changes in the lipid profile

VO_2max_: maximal oxygen consumption. 1 RM: one repetition maximal. TG: triglycerides. FFA: free fatty acid. VLDL-TG: very low density lipoprotein. NEFA: non-esterified fatty acids. LDL: low density lipoprotein. HDL: high density lipoprotein. MTP: microsomal transfer protein. apoB: apolipoprotein B.

## References

[1] Adams JH, Koeslag JH (1989). Post-exercise ketosis and the glycogen content of liver and muscle in rats on a high carbohydrate diet. *European Journal of Applied Physiology and Occupational Physiology*.

[2] Aellen R, Hollmann W, Boutellier U (1993). Effects of aerobic and anaerobic training on plasma lipoproteins. *International Journal of Sports Medicine*.

[3] Olofsson SO, Boström P, Andersson L, Rutberg M, Perman J, Borén J (2009). Lipid droplets as dynamic organelles connecting storage and efflux of lipids. *Biochimica et Biophysica Acta*.

[4] Badman MK, Pissios P, Kennedy AR, Koukos G, Flier JS, Maratos-Flier E (2007). Hepatic fibroblast growth factor 21 is regulated by PPAR*α* and is a key mediator of hepatic lipid metabolism in ketotic states. *Cell Metabolism*.

[5] Beattie MA, Winder WW (1985). Attenuation of postexercise ketosis in fasted endurance-trained rats. *The American Journal of Physiology*.

[6] Gibbons GF, Wiggins D, Brown AM, Hebbachi AM (2004). Synthesis and function of hepatic very-low-density lipoprotein. *Biochemical Society Transactions*.

[7] Gibbons GF, Brown AM, Wiggins D, Pease R (2002). The roles of insulin and fatty acids in the regulation of hepatic very-low-density lipoprotein assembly. *Journal of the Royal Society of Medicine*.

[8] Lira FS, Rosa JC, Lima-Silva AE (2010). Sedentary subjects have higher PAI-1 and lipoproteins levels than highly trained athletes. *Diabetology and Metabolic Syndrome*.

[9] Magkos F, Lavoie JM, Kantartzis K, Gastaldelli A (2012). Diet and exercise in the treatment of Fatty liver. *Journal of Nutrition and Metabolism*.

[10] Belmonte MA, Aoki MS, Tavares FL, Seelaender MCL (2004). Rat myocellular and perimysial intramuscular triacylglycerol: a histological approach. *Medicine and Science in Sports and Exercise*.

[11] Lira FS, Tavares FL, Yamashita AS (2008). Effect of endurance training upon lipid metabolism in the liver of cachectic tumour-bearing rats. *Cell Biochemistry and Function*.

[12] Chapados NA, Seelaender M, Levy E, Lavoie JM (2009). Effects of exercise training on hepatic microsomal triglyceride transfer protein content in rats. *Hormone and Metabolic Research*.

[13] Garber CE, Blissmer B, Deschenes MR (2011). Quantity and quality of exercise for developing and maintaining cardiorespiratory, musculoskeletal, and neuromotor fitness in apparently healthy adults: guidance for prescribing exercise. *Medicine and Science in Sports and Exercise*.

[14] Lira FS, Zanchi NE, Lima-Silva AE (2009). Acute high-intensity exercise with low energy expenditure reduced LDL-c and total cholesterol in men. *European Journal of Applied Physiology*.

[15] Magkos F, Tsekouras YE, Prentzas KI (2008). Acute exercise-induced changes in basal VLDL-triglyceride kinetics leading to hypotriglyceridemia manifest more readily after resistance than endurance exercise. *Journal of Applied Physiology*.

[16] Gibala MJ (2007). High-intensity interval training: a time-efficient strategy for health promotion?. *Current Sports Medicine Reports*.

[17] Stein RA, Michielli DW, Glantz MD (1990). Effects of different exercise training intensities on lipoprotein cholesterol fractions in healthy middle-aged men. *American Heart Journal*.

[18] Ferguson MA, Alderson NL, Trost SG, Essig DA, Burke JR, Durstine JL (1998). Effects of four different single exercise sessions on lipids, lipoproteins, and lipoprotein lipase. *Journal of Applied Physiology*.

[19] Tsekouras YE, Magkos F, Kellas Y, Basioukas KN, Kavouras SA, Sidossis LS (2008). High-intensity interval aerobic training reduces hepatic very low-density lipoprotein-triglyceride secretion rate in men. *American Journal of Physiology-Endocrinology and Metabolism*.

[20] Maraki M, Christodoulou N, Aggelopoulou N (2009). Exercise of low energy expenditure along with mild energy intake restriction acutely reduces fasting and postprandial triacylglycerolaemia in young women. *British Journal of Nutrition*.

[21] Lira FS, Zanchi NE, Lima-Silva AE (2010). Is acute supramaximal exercise capable of modulating lipoprotein profile in healthy men?. *European Journal of Clinical Investigation*.

[22] Mondon CE, Dolkas CB, Tobey T, Reaven GM (1984). Causes of triglyceride-lowering effect of exercise training in rats. *Journal of Applied Physiology Respiratory Environmental and Exercise Physiology*.

[23] Wallace MB, Moffatt RJ, Haymes EM, Green NR (1991). Acute effects of resistance exercise on parameters of lipoprotein metabolism. *Medicine and Science in Sports and Exercise*.

[24] Tsekouras YE, Magkos F, Prentzas KI (2009). A single bout of whole-body resistance exercise augments basal VLDL-triacylglycerol removal from plasma in healthy untrained men. *Clinical Science*.

[25] Lira FS, Yamashita AS, Uchida MC (2010). Low and moderate, rather than high intensity strength exercise induces benefit regarding plasma lipid profile. *Diabetology and Metabolic Syndrome*.

[26] Magkos F, Tsekouras YE, Prentzas KI (2008). Acute exercise-induced changes in basal VLDL-triglyceride kinetics leading to hypotriglyceridemia manifest more readily after resistance than endurance exercise. *Journal of Applied Physiology*.

[27] Magkos F, Patterson BW, Mohammed BS, Mittendorfer B (2007). A single 1-h bout of evening exercise increases basal FFA flux without affecting VLDL-triglyceride and VLDL-apolipoprotein B-100 kinetics in untrained lean men. *American Journal of Physiology-Endocrinology and Metabolism*.

[28] Seip RL, Semenkovich CF (1998). Skeletal muscle lipoprotein lipase: molecular regulation and physiological effects in relation to exercise. *Exercise and Sport Sciences Reviews*.

[29] Pilegaard H, Ordway GA, Saltin B, Neufer PD (2000). Transcriptional regulation of gene expression in human skeletal muscle during recovery from exercise. *American Journal of Physiology-Endocrinology and Metabolism*.

[30] Seip RL, Mair K, Cole TG, Semenkovich CF (1997). Induction of human skeletal muscle lipoprotein lipase gene expression by short-term exercise is transient. *American Journal of Physiology-Endocrinology and Metabolism*.

[31] Fisher RM, Coppack SW, Humphreys SM, Gibbons GF, Frayn KN (1995). Human triacylglycerol-rich lipoprotein subfractions as substrates for lipoprotein lipase. *Clinica Chimica Acta*.

[32] Perseghin G, Lattuada G, De Cobelli F (2007). Habitual physical activity is associated with intrahepatic fat content in humans. *Diabetes Care*.

[33] Gauthier MS, Couturier K, Latour JG, Lavoie JM (2003). Concurrent exercise prevents high-fat-diet-induced macrovesicular hepatic steatosis. *Journal of Applied Physiology*.

[34] Rector RS, Thyfault JP, Morris RT (2008). Daily exercise increases hepatic fatty acid oxidation and prevents steatosis in Otsuka Long-Evans Tokushima Fatty rats. *American Journal of Physiology*.

[35] Yasari S, Prud’Homme D, Wang D (2010). Exercise training decreases hepatic SCD-1 gene expression and protein content in rats. *Molecular and Cellular Biochemistry*.

[36] Musso G, Gambino R, Cassader M (2009). Recent insights into hepatic lipid metabolism in non-alcoholic fatty liver disease (NAFLD). *Progress in Lipid Research*.

[37] Lira FS, Yamashita AS, Carnevalli JRL (2010). Exercise training reduces PGE2 levels and restores steatosis hepatic in tumour-bearing rats. *Hormone and Metabolic Research*.

[38] Rector SR, Thyfault JP, Laye MJ (2008). Cessation of daily exercise dramatically alters precursors of hepatic steatosis in Otsuka Long-Evans Tokushima Fatty (OLETF) rats. *Journal of Physiology*.

[39] McGarry JD, Foster DW (1980). Regulation of hepatic fatty acid oxidation and ketone body production. *Annual Review of Biochemistry*.

[40] Hardie DG (1989). Regulation of fatty acid synthesis via phosphorylation of acetyl-CoA carboxylase. *Progress in Lipid Research*.

[41] Ruderman NB, Park H, Kaushik VK (2003). AMPK as a metabolic switch in rat muscle, liver and adipose tissue after exercise. *Acta Physiologica Scandinavica*.

[42] Hardie DG, Corton J, Ching YP, Davies SP, Hawley S (1997). Regulation of lipid metabolism by the AMP-activated protein kinase. *Biochemical Society Transactions*.

[43] Berglund ED, Kang L, Lee-Young RS (2010). Glucagon and lipid interactions in the regulation of hepatic AMPK signaling and expression of PPAR*α* and FGF21 transcripts in vivo. *American Journal of Physiology-Endocrinology and Metabolism*.

[44] Hoene M, Weigert C (2010). The stress response of the liver to physical exercise. *Exercise Immunology Review*.

[45] Hoene M, Lehmann R, Hennige AM (2009). Acute regulation of metabolic genes and insulin receptor substrates in the liver of mice by one single bout of treadmill exercise. *Journal of Physiology*.

[46] Felig P, Wahren J (1975). Fuel homeostasis in exercise. *New England Journal of Medicine*.

[47] Galbo H, Holst JJ, Christensen NJ (1979). The effect of different diets and of insulin on the hormonal response to prolonged exercise. *Acta Physiologica Scandinavica*.

[48] Noland RC, Thyfault JP, Henes ST (2007). Artificial selection for high-capacity endurance running is protective against high-fat diet-induced insulin resistance. *American Journal of Physiology-Endocrinology and Metabolism*.

[49] Wasserman DH, O’Doherty RM, Zinker BA (1995). Role of the endocrine pancreas in control of fuel metabolism by the liver during exercise. *International Journal of Obesity*.

[50] Wasserman DH, Cherrington AD (1991). Hepatic fuel metabolism during muscular work: role and regulation. *American Journal of Physiology-Endocrinology and Metabolism*.

[51] Drouin R, Lavoie C, Bourque J, Ducros F, Poisson D, Chiasson JL (1998). Increased hepatic glucose production response to glucagon in trained subjects. *American Journal of Physiology-Endocrinology and Metabolism*.

[52] Légaré A, Drouin R, Milot M (2001). Increased density of glucagon receptors in liver from endurance-trained rats. *American Journal of Physiology-Endocrinology and Metabolism*.

[53] Marliss EB, Simantirakis E, Miles PDG (1991). Glucoregulatory and hormonal responses to repeated bouts of intense exercise in normal male subjects. *Journal of Applied Physiology*.

[54] Galbo H (1985). The hormonal response to exercise. *Proceedings of the Nutrition Society*.

[55] Deibert DC, DeFronzo RA (1980). Epinephrine-induced insulin resistance in man. *Journal of Clinical Investigation*.

[56] Saccà L, Eigler N, Cryer PE, Sherwin RS (1979). Insulin antagonistic effects of epinephrine and glucagon in the dog. *The American Journal of Physiology*.

[57] Marker JC, Arnall DA, Conlee RK, Winder WW (1986). Effect of adrenodemedullation on metabolic responses to high-intensity exercise. *The American Journal of Physiology*.

[58] Kjaer M, Engfred K, Fernandes A, Secher NH, Galbo H (1993). Regulation of hepatic glucose production during exercise in humans: role of sympathoadrenergic activity. *American Journal of Physiology-Endocrinology and Metabolism*.

[59] Kjaer M, Engered K, Galbo H (1991). Hepatic glucose production during exercise in liver-transplanted subjects. *Scandinavian Journal of Gastroenterology*.

[60] Berglund ED, Lee-Young RS, Lustig DG (2009). Hepatic energy state is regulated by glucagon receptor signaling in mice. *Journal of Clinical Investigation*.

[61] Lavoie JM (2002). The contribution of afferent signals from the liver to metabolic regulation during exercise. *Canadian Journal of Physiology and Pharmacology*.

[62] Longuet C, Sinclair EM, Maida A (2008). The glucagon receptor is required for the adaptive metabolic response to fasting. *Cell Metabolism*.

[63] Christensen NJ, Galbo H (1983). Sympathetic nervous activity during exercise. *Annual Review of Physiology*.

[64] Van Dijk G, Balkan B, Lindfeldt J (1994). Contribution of liver nerves, glucagon, and adrenaline to the glycaemic response to exercise in rats. *Acta Physiologica Scandinavica*.

[65] McMurray RG, Forsythe WA, Mar MH, Hardy CJ (1987). Exercise intensity-related responses of *β*-endorphin and catecholamines. *Medicine and Science in Sports and Exercise*.

[66] Tavares FL, Seelaender MCL (2008). Hepatic denervation impairs the assembly and secretion of VLDL-TAG. *Cell Biochemistry and Function*.

